# Transcriptome-Wide Analysis of N6-Methyladenosine-Modified Long Noncoding RNAs in Particulate Matter-Induced Lung Injury

**DOI:** 10.3390/toxics13020098

**Published:** 2025-01-27

**Authors:** Yingying Zeng, Guiping Zhu, Wenjun Peng, Hui Cai, Chong Lu, Ling Ye, Meiling Jin, Jian Wang

**Affiliations:** 1 Department of Pulmonary Medicine, Zhongshan Hospital, Fudan University, Shanghai 200030, China; ying.respire@foxmail.com (Y.Z.); gpzhu0813@163.com (G.Z.); wjpeng1126@163.com (W.P.); cai416834096@126.com (H.C.); 18717726980@163.com (C.L.); 2 Department of Allergy, Zhongshan Hospital, Fudan University, Shanghai 200030, China; ye.ling@zs-hospital.sh.cn

**Keywords:** long noncoding RNAs, m^6^A methylation, particulate matter, lung injury

## Abstract

Background: N6-methyladenosine (m^6^A) modification plays a crucial role in the regulation of diverse cellular processes influenced by environmental factors. Nevertheless, the involvement of m^6^A-modified long noncoding RNAs (lncRNAs) in the pathogenesis of lung injury induced by particulate matter (PM) remains largely unexplored. Methods: Here, we establish a mouse model of PM-induced lung injury. We utilized m6A-modified RNA immunoprecipitation sequencing (MeRIP-seq) to identify differentially expressed m6A peaks on long non-coding RNAs (lncRNAs). Concurrently, we performed lncRNA sequencing (lncRNA-seq) to determine the differentially expressed lncRNAs. The candidate m6A-modified lncRNAs in the lung tissues of mice were identified through the intersection of the data obtained from these two sequencing approaches. Results: A total of 664 hypermethylated m^6^A peaks on 644 lncRNAs and 367 hypomethylated m^6^A peaks on 358 lncRNAs are confirmed. We use bioinformatic tools to analyze the potential functions and pathways of these m^6^A-modified lncRNAs, revealing their involvement in regulating inflammation, immune response, and metabolism-related pathways. Three key m^6^A-modified lncRNAs (lncRNA NR_003508, lncRNA uc008scb.1, and lncRNA ENSMUST00000159072) are identified through a joint analysis of the MeRIP-seq and lncRNA-seq data, and their validation is carried out using MeRIP-PCR and qRT-PCR. Analysis of the coding-non-coding gene co-expression network reveals that m^6^A-modified lncRNAs NR_003508 and uc008scb.1 participate in regulating pathways associated with inflammation and immune response. Conclusions: This study first provides a comprehensive transcriptome-wide analysis of m^6^A methylation profiling in lncRNAs associated with PM-induced lung injury and identifies three pivotal candidate m^6^A-modified lncRNAs. These findings shed light on a novel regulatory mechanism underlying PM-induced lung injury.

## 1. Introduction

Air pollution is widely recognized as a major risk factor that significantly impacts human health. In 2019, more than 90% of the global population suffered from particulate matter (PM) with an aerodynamic diameter ≤ 2.5 μm (PM2.5) pollution. Short-term PM exposure has been linked to a significant increase in the daily mortality associated with respiratory diseases in more than 600 cities globally [1]. Furthermore, PM exposure has been linked to various respiratory diseases, particularly chronic airway diseases, including bronchial asthma and chronic obstructive pulmonary disease (COPD). According to the China Pulmonary Health Study, exposure to PM2.5 has been identified as a significant risk factor contributing to the prevalence of COPD and bronchial asthma in the Chinese adult population [2,3]. Different biological mechanisms may contribute to PM-induced lung injury. Our previous research has demonstrated the crucial involvement of inflammatory responses and oxidative stress in the development of lung injury caused by exposure to PM [4]. Multiple pro-inflammatory proteins, circRNAs, and microRNAs have been identified as contributors to the exacerbation of PM-induced airway inflammation through the regulation of signaling pathways, such as MAPK, NF-κB, and PI3K-AKT [5,6,7,8]. However, the underlying mechanism behind these findings remains to be fully understood and requires further investigation.

N6-Methyladenosine (m^6^A) is a prominent and highly prevalent epigenetic modification of RNA in mammalian systems. The m^6^A modification is a dynamically reversible process, which is modulated by methylases and demethylases. This modification plays a crucial role in governing various aspects of RNA biology, including RNA stability, degradation, splicing, and translation. The change of m^6^A methylation levels in RNAs has been linked to a wide range of pathological processes, including cancer development, inflammation, lipid metabolism, and DNA damage repair [9]. A growing body of research has demonstrated the association between m^6^A modification and the adverse effects caused by PM. PM exposure can induce an elevation in m^6^A methylation levels, promoting mucus production and lung inflammation [10]. Furthermore, our previous research has indicated that PM exposure leads to an elevated m^6^A methylation level of interleukin-1 alpha (IL-1a) mRNA, triggering airway inflammation via the MAPK signaling pathway [11].

Long noncoding RNAs (lncRNAs), characterized by a length exceeding 200 nucleotides (nt), dominate the landscape of noncoding RNAs (ncRNAs) and exert a pivotal function in the regulation of transcription, post-translational regulation, and chromatin architecture [12,13]. Emerging evidence suggests that numerous lncRNAs are intricately involved in the development of cardiopulmonary diseases induced by PM exposure [14,15]. Furthermore, m^6^A modification could regulate the expression of lncRNAs in different diseases. Recent studies have demonstrated that m^6^A-modified lncRNAs promote lung damage, lung cancer development, and metastasis [16,17]. Despite advancements in the field of m^6^A modification research, the precise association between m^6^A methylation and the expression of lncRNAs in PM-induced lung injury remains poorly understood. Here, we employed m^6^A-modified RNA immunoprecipitation sequencing (MeRIP-seq) to comprehensively characterize the differentially expressed m^6^A-modified lncRNAs in the lung tissues of mice exposed to PM. Three key m^6^A-modified lncRNAs (lncRNA NR_003508, lncRNA uc008scb.1, and lncRNA ENSMUST00000159072) have been identified as being involved in the development of lung injury induced by PM exposure. Our findings provide a novel insight into the epigenetic mechanisms underlying PM-induced lung injury.

## 2. Materials and Methods

### 2.1. Animals Experiment

We implemented the PM-exposed mice model by employing the experimental procedures outlined in our previous study [18,19]. In brief, six- to eight-week-old female C57BL/6 mice were randomly assigned to two groups, with each group consisting of three mice. The PM exposed group of mice were subjected to daily intratracheal injections of a 25 μL suspension containing 100 μg PM, while the control group received daily intratracheal injections of 25 μL sterile PBS. This administration was performed consecutively for two days, and on the third day, all mice were sacrificed. The mice were anesthetized by intraperitoneal (IP) injection of tribromoethanol (Avertin) at a dosage 250 mg/kg. MeRIP-seq and lncRNA sequencing were performed on the right lung tissues, while the left lung tissues were reserved for validation purposes. The lung tissues were stored at −80 °C before use. PM (SRM 1649b) was purchased from the National Institute of Standards and Technology (NIST). Detailed information about the components of PM can be found on the NIST website (www.nist.gov, accessed on 10 March 2023). The study was conducted according to the guidelines of the Declaration of Helsinki and approved by the Animal Care and Use Committee of Zhongshan Hospital, Fudan University.

### 2.2. MeRIP Sequencing and Identification of Differentially Methylated lncRNAs

Lung tissue RNA was isolated using TRIzol and quantified using a NanoDrop ND-1000 spectrophotometer (Thermo Fisher Scientific, Waltham, MA, USA). Eligible RNA samples for MeRIP-seq should exhibit high purity, as evidenced by an optical density (OD) ratio of 260/280 ranging from 1.8 to 2.1, and intactness, which is confirmed by the presence of distinct 18S and 28S ribosomal RNA bands on agarose gel electrophoresis, with the 28S band being approximately twice as intense as the 18S band. Subsequently, the eligible RNA samples were prepared for MeRIP-seq by Cloud-Seq Biotech (Shanghai, China). First, m^6^A-modified RNA was enriched through m^6^A RNA immunoprecipitation (IP) using the GenSeq^TM^ m^6^A-MeRIP kit (GenSeq Inc., Shanghai, China) as per the provided instructions. Next, the enriched RNA samples and control samples were subjected to create RNA sequencing libraries. After evaluating the quality of the libraries, they were sequenced on an Illumina NovaSeq 6000 instrument using 150 bp paired-end reads.

After a quality control process using Q30 and removal of 3′ adaptors and low-quality reads using cutadapt software (v1.9.3), the eligible paired-end reads were then aligned to the UCSC MM10 reference genome using Hisat2 software (v2.0.4). MACS software (v2.1.1) was utilized to detect the methylated sites on the RNAs. Differential methylation analysis of lncRNAs between the PM and control groups was conducted using DiffReps software (v1.55.6). The analysis applied stringent criteria, including a *p*-value < 0.000001 and fold change ≥ 2, to identify differentially methylated sites. Motifs enriched in m6A peaks within all lncRNAs were identified using HOMER software (http://homer.ucsd.edu/homer/, accessed on 15 March 2023). Subsequently, Gene Ontology (GO) and Kyoto Encyclopedia of Genes and Genomes (KEGG) analyses were conducted to investigate the functions and pathways of these associated genes. Finally, the DMS alignment was visualized using Integrative Genomics Viewer (IGV, www.broadinstitute.org/igv, accessed on 16 March 2023) in accordance with the manufacturer’ s instructions.

### 2.3. LncRNA Sequencing

RNA extraction and quality control procedures were conducted using the aforementioned protocol. LncRNA sequencing was also carried out by Cloud-Seq Biotech. Briefly, following the depletion of rRNA from total RNA samples, RNA libraries were generated using the NEBNext^®^ UltraTM II Directional RNA Library Prep Kit (New England Biolabs) and quality control was performed using the BioAnalyzer 2100 system (Agilent Technologies, Inc., Santa Clara, CA, USA). Finally, lncRNA sequencing was carried out on an Illumina NovaSeq 6000.

After quality control using Q30 and removal of 3′ adaptors and low-quality reads using cutadapt software (v1.9.3), the eligible reads were then aligned to the UCSC MM10 reference genome using Hisat2 software (v2.0.4). The FPKM expression profiles of lncRNAs were generated by employing cuffdiff software (v2.2.1, part of cufflinks), with guidance from the Ensembl gtf gene annotation file. Then, the FPKM values were utilized for calculating the fold change and *p*-value. The differentially expressed lncRNAs were identified by applying the criteria of *p*-value < 0.05 and fold change ≥ 2. The prediction of target genes for lncRNAs was based on their proximity to neighboring genes. In addition, GO and KEGG analyses were performed on the identified target genes to gain a comprehensive understanding of their biological functions and associated pathways.

### 2.4. Real-Time qPCR (RT-qPCR)

RNA extraction and subsequent quality control procedures were performed following the aforementioned protocol. The cDNA synthesis and RT-qPCR analysis were performed using reagents from TaKaRa Bio following the protocol established in our previous study [11]. GAPDH was utilized as the reliable internal reference gene for normalization purposes. The relative gene expression levels were determined using the established 2^−ΔΔCt^ method, ensuring a precise and accurate assessment of transcriptional changes.

### 2.5. MeRIP-qPCR

An MeRIP-qPCR assay was conducted to verify the m^6^A peaks of lncRNAs using the GenSeq^®^ m^6^A-MeRIP kit following the manufacturer’s protocols. Briefly, RNA was incubated with m^6^A or IgG antibodies coupled to magnetic beads. Then, the elution of the immunoprecipitated m^6^A-modified RNA from the magnetic beads was carried out in accordance with the manufacturer’s instructions. The input samples and IP eluents were then subjected to RT-qPCR analysis.

### 2.6. Data Analysis

The results of MeRIP-qPCR and RT-qPCR were compared using a two-tailed unpaired Student’ s *t*-test and statistical significance was defined as a *p*-value less than 0.05 in the two groups. Data were presented as the mean ± SEM and analyzed using SPSS v19.0 software (IBM Corp., Armonk, NY, USA).

## 3. Results

### 3.1. Characteristics of m^6^A Peaks on lncRNAs in Lung Tissues of Mice Exposed to PM

**Figure 1:**
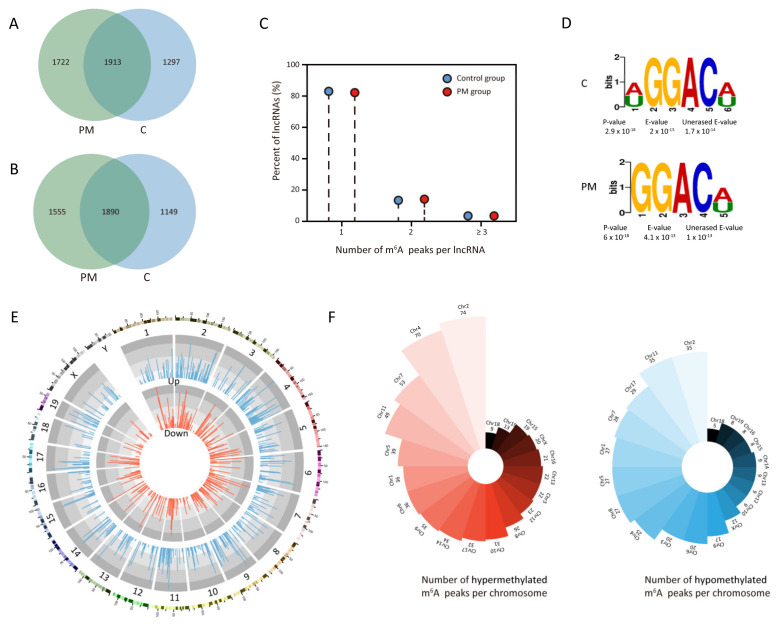
Overview of m^6^A modification on lncRNAs in lung tissues of mice with or without PM exposure. (**A**) Venn diagram showing the numbers of unique and common m^6^A peaks on lncRNAs. (**B**) Venn diagram showing the numbers of unique and common m^6^A-modified lncRNAs. (**C**) Proportions of lncRNAs containing varying numbers of m^6^A peaks in both groups. (**D**) The motif enriched from m^6^A peaks in both groups. (**E**) Circos plot showing the distribution of differentially hypermethylated and hypomethylated m^6^A peaks in different chromosome. (**F**) Polar bar diagram displaying the number of hypotheylated and hypermethylated m^6^A peaks per chromosome in two groups. PM, particulate matter.

MeRIP-seq was used to uncover the features of m^6^A-modified sites on lncRNAs in the lung tissues of mice with or without PM exposure. In total, we identified 3635 m^6^A peaks on 3445 lncRNAs in the lung tissues of mice exposed to PM, and 3210 m^6^A peaks on 3039 lncRNAs in the lung tissues of control mice. Of these, 1913 m^6^A peaks on 1890 lncRNAs were co-expressed in both groups (Figure 1A,B). Further analysis revealed that over 80% of the m^6^A-modified lncRNAs in both groups had one or two m^6^A peaks each (Figure 1C). We used the HOMER software to analyze the presence of the conserved RRACH sequence within m^6^A peaks, revealing that the GGACU/GGACA motif was predominantly enriched in both groups (Figure 1D).

In the PM-exposed group, we found 664 m^6^A peaks hypermethylated on 644 lncRNAs and 367 m^6^A peaks hypomethylated on 358 lncRNAs in comparison to the control group. The top 10 differentially hypermethylated and hypomethylated m^6^A peaks are shown in Table 1. The mapping of these m^6^A peaks on lncRNAs to different chromosomes was visually displayed in Figure 1E. We found that chromosome 2 had the most hypermethylated m^6^A peaks, with chromosomes 4, 7, 11, and 5 following in descending order. Meanwhile, chromosome 2 also had the most hypomethylated m^6^A peaks, followed by chromosomes 11, 17, 7, and 1 (Figure 1F). These findings indicate that PM exposure significantly alters the m^6^A modification of lncRNAs in the lung tissues of mice.

### 3.2. Function Enrichment and Pathway Analysis of m^6^A-Modified lncRNAs

**Figure 2:**
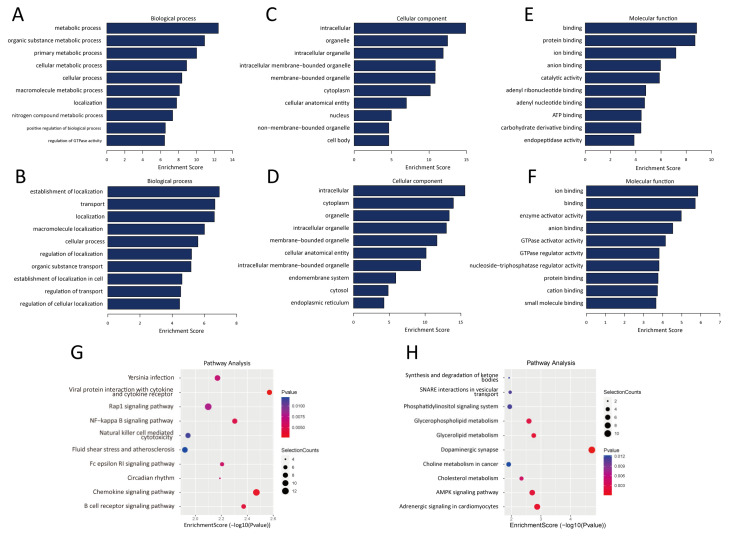
GO enrichment and KEGG analysis of lncRNAs with differentially methylated m^6^A peaks. (**A**) GO enrichment of lncRNAs with differentially hypermethylated m^6^A peaks in the biological process category. (**B**) GO enrichment of lncRNAs with differentially hypomethylated m^6^A peaks in the biological process category. (**C**) GO enrichment of lncRNAs with differentially hypermethylated m^6^A peaks in the cellular component category. (**D**) GO enrichment of lncRNAs with differentially hypomethylated m^6^A peaks in the cellular component category. (**E**) GO enrichment of lncRNAs with differentially hypermethylated m^6^A peaks in the molecular function category. (**F**) GO enrichment of lncRNAs with differentially hypomethylated m^6^A peaks in the molecular function category. (**G**) KEGG analysis of lncRNAs with differentially hypermethylated m^6^A peaks. (**H**) KEGG analysis of lncRNAs with differentially hypomethylated m^6^A peaks. GO, Gene Ontology. KEGG, Kyoto Encyclopedia of Genes and Genomes.

GO analysis was conducted to predict the potential functions of the differentially m^6^A-modified lncRNAs in the lung tissues of mice exposed to PM. In the biological process category, lncRNAs with m^6^A hypermethylation were enriched in metabolic processes, organic substance metabolic processes, primary metabolic processes, cellular metabolic processes, and cellular processes (Figure 2A), whereas lncRNAs with m^6^A hypomethylation were enriched in the establishment of localization, transport, localization, macromolecule localization, and cellular processes (Figure 2B). In the cellular component category, lncRNAs with m^6^A hypermethylation were enriched in intracellular, organelle, intracellular organelle, and membrane-bounded organelle (Figure 2C), whereas lncRNAs with m^6^A hypomethylation were enriched in intracellular, cytoplasmic, organelle, intracellular organelle, and membrane-bounded organelle (Figure 2D). In the molecular function category, lncRNAs with m^6^A hypermethylation were enriched in binding, protein binding, ion binding, anion binding, and catalytic activity (Figure 2E), whereas lncRNAs with m^6^A hypomethylation were enriched in ion binding, binding, enzyme activator activity, anion binding, and GTPase activator activity (Figure 2F).

Next, we used KEGG analysis to identify the pathways enriched in m^6^A-modified lncRNAs. LncRNAs with m^6^A hypermethylation were enriched in several important inflammatory and immune pathways, including the NF-κB signaling pathway, chemokine signaling pathway, B-cell receptor signaling pathway, and natural killer cell-mediated cytotoxicity (Figure 2G). Furthermore, lncRNAs with m^6^A hypomethylation were involved in regulating the AMPK signaling pathway, glycerolipid metabolism, glycerophospholipid metabolism, and cholesterol metabolism (Figure 2H). These results show that m^6^A-modified lncRNAs play a significant role in regulating lung damage caused by PM exposure.

### 3.3. Differential Expression and Functional Analysis of lncRNAs in Lung Tissues of Mice Exposed to PM

To investigate the expression profiles of lncRNAs in lung tissues of mice exposed to PM, we analyzed the m^6^A input library to identify significantly expressed lncRNAs. A total of 227 lncRNAs showed differential expression in the lung tissues of PM-exposed mice, with 93 upregulated and 134 downregulated. Then, GO enrichment and KEGG analysis were conducted to predict the role of lncRNAs in PM-induced lung damage. In the biological process category, upregulated lncRNAs were enriched in cellular metabolic processes, primary metabolic processes, and nucleic acid metabolic processes, whereas downregulated lncRNAs were involved in negative regulation of cellular biosynthetic processes, negative regulation of biosynthetic processes, and negative regulation of transcription by RNA polymerase II. For the cellular component category, upregulated lncRNAs were enriched in the intracellular organelle lumen, organelle lumen, and membrane-enclosed lumen, while downregulated lncRNAs were involved in intracellular organelles, membrane-bound organelles, and organelles. For the molecular function category, upregulated lncRNAs were enriched in enzyme binding, transferase activity, and nucleic acid binding, while downregulated lncRNAs were involved in binding, organic cyclic compound binding, and heterocyclic compound binding. Additionally, KEGG analysis showed that upregulated lncRNAs were enriched in glutathione metabolism, fluid shear stress, atherosclerosis, and mismatch repair, while downregulated lncRNAs were linked to the AMPK signaling pathway, mRNA surveillance pathway, and endocytosis. The results of the GO enrichment and KEGG analysis were presented in Supplementary Figure S1.

### 3.4. Conjoint Analysis of MeRIP-Seq and lncRNA-Seq Data

**Figure 3:**
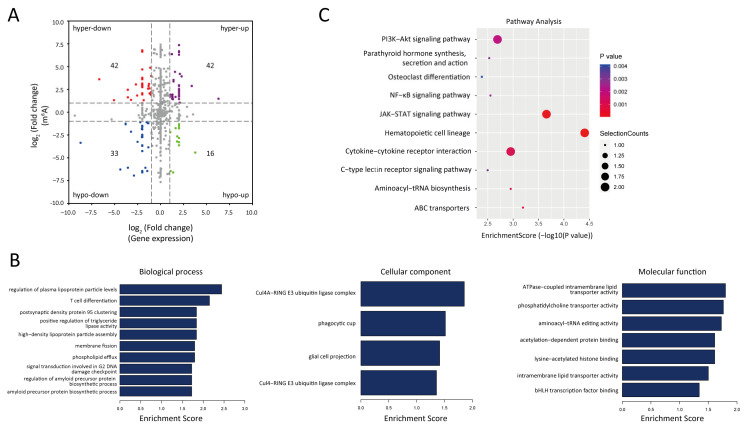
Conjoint analysis of MeRIP-seq and lncRNA-seq data. (**A**) Four-quadrant graph showing the lncRNAs with differentially methylated m^6^A peaks. The red dots represent the downregulated lncRNAs with m^6^A hypermethylation, the purple dots represent the upregulated lncRNAs with m^6^A hypermethylation, the blue dots represent downregulated lncRNAs with m^6^A hypomethylation, and the green dots represent upregulated lncRNAs with m^6^A hypomethylation. (**B**) GO analysis of the lncRNAs harboring differentially methylated m^6^A peaks. (**C**) KEGG analysis of the lncRNAs harboring differentially methylated m^6^A peaks. GO, Gene Ontology. KEGG, Kyoto Encyclopedia of Genes and Genomes.

To further explore the regulatory role of m^6^A modification in the expression of lncRNAs in PM-exposed mice, we performed an integrative analysis of MeRIP-seq and lncRNA-seq data. A total of 42 upregulated lncRNAs with m^6^A hypermethylation (hyper-up), 16 upregulated lncRNAs with m^6^A hypomethylation (hypo-up), 42 downregulated lncRNAs with m^6^A hypermethylation (hyper-down), and 33 downregulated lncRNAs with m^6^A hypomethylation (hypo-down) were identified (Figure 3A). Next, we performed GO and KEGG analysis to predict the potential biological functions of the 143 lncRNAs with differentially expressed m^6^A peaks. The GO enrichment terms for these lncRNAs are shown in Figure 3B. The KEGG analysis showed several important inflammatory pathways, including the JAK-STAT signaling pathway, PI3K-AKT signaling pathway, NF-κB signaling pathway, and cytokine-cytokine receptor interaction (Figure 3C). These findings suggest that m^6^A modification may control the expression of lncRNAs to regulate PM-induced lung damage.

### 3.5. Construction of m^6^A-Modified lncRNAs-mRNAs Network

**Figure 4:**
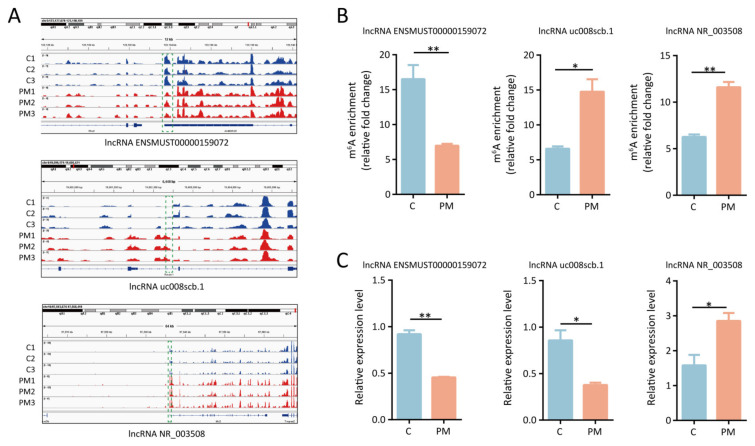
The validation of three m^6^A-modified lncRNAs in the lung tissues from PM-exposed mice. (**A**) Visualization of m^6^A peaks on lncRNA NR_003508, lncRNA uc008scb.1, and lncRNA ENSMUST00000159072 in lung tissues of mice with or without PM exposure. (**B**) MeRIP-qPCR indicating the level of m^6^A modification on lncRNA NR_003508, lncRNA uc008scb.1, and lncRNA ENSMUST00000159072 in lung tissues of PM-exposed mice. (**C**) RT-qPCR showing the relative expression of lncRNA NR_003508, lncRNA uc008scb.1, and lncRNA ENSMUST00000159072 in lung tissues of mice exposed to PM. Values represent mean ± SEM; *, *p* < 0.05, **, *p* < 0.01, compared with the control group, n = 3. PM, particulate matter.

To validate the reliability of the MeRIP-seq data, three differentially expressed lncRNAs with m^6^A modification (lncRNA NR_003508, lncRNA uc008scb.1, and lncRNA ENSMUST00000159072) were selected for further validation. The visualization of m^6^A peaks on lncRNA NR_003508, lncRNA uc008scb.1, and lncRNA ENSMUST00000159072 are shown in Figure 4A. In line with the MeRIP-seq data, MeRIP-qPCR analysis revealed that PM exposure decreased m^6^A enrichment in lncRNA ENSMUST00000159072 and increased m^6^A enrichment in lncRNA NR_003508 and lncRNA uc008scb.1 in mice lung tissues (Figure 4B). Furthermore, RT-qPCR analysis confirmed that PM exposure significantly increased the expression of lncRNA NR_003508 while decreasing the expression of lncRNA uc008scb.1 and lncRNA ENSMUST00000159072 in lung tissues, in line with the lncRNA-seq data (Figure 4C).

**Figure 5:**
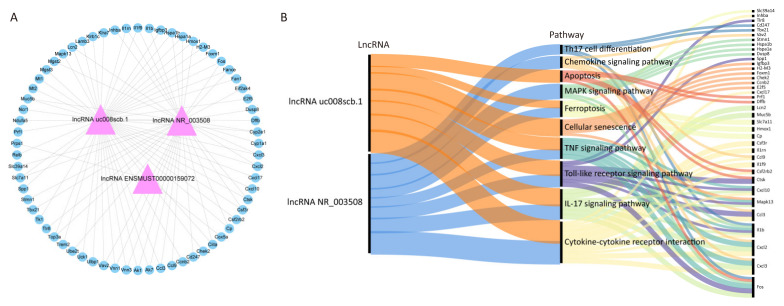
Construction of lncRNA-mRNA co-expression network for three m^6^A-modified lncRNAs. (**A**) The lncRNA-mRNA co-expression network of three lncRNAs (lncRNA NR_003508, lncRNA uc008scb.1, and lncRNA ENSMUST00000159072) and their co-expressed mRNAs. (**B**) The Sankey diagram showing the potential pathways regulated by lncRNA NR_003508 and lncRNA uc008scb.1, along with their co-expressed mRNAs.

To investigate the potential biological functions of these three lncRNAs in PM-exposed lung tissues, we correlated their expression with mRNA expression profiles from our previous study on PM-exposed lung tissues [12]. We constructed a coding-non-coding gene co-expression (CNC) network linking the three lncRNAs with the co-expressed mRNAs in PM-exposed lung tissues (Figure 5A). The potential functions of these co-expressed mRNAs were further elucidated using GO and KEGG analysis. The results of GO and KEGG analysis were shown in Supplementary Figure S2. As for the limited number of lncRNA ENSMUST00000159072-related mRNAs, pathway enrichment of the lncRNAs (lncRNA NR_003508 and lncRNA uc008scb.1) and their co-expressed mRNAs was performed. The Sankey diagram showed that lncRNA NR_003508 and lncRNA uc008scb.1 were involved in regulating several key pathways, including Th17 cell differentiation, chemokine signaling, apoptosis, MAPK signaling, ferroptosis, cellular senescence, TNF signaling, Toll-like receptor signaling, IL-17 signaling, and cytokine-cytokine receptor interaction (Figure 5B). These findings indicate that the three lncRNAs play a critical role in the pathogenesis of PM-induced lung damage.

## 4. Discussion

LncRNA has been recognized as a pivotal member of the noncoding RNA family, playing a significant role in the development of various lung diseases. Here, we presented the overall expression patterns of m^6^A-modified lncRNAs in lung tissues of mice with and without PM exposure. Through the conjoint analysis of MeRIP-seq and lncRNA-seq data, we identified m^6^A-modified lncRNAs that regulate key inflammatory, immune, and metabolic pathways in lung tissues of mice exposed to PM. Furthermore, three key m^6^A-modified lncRNAs (lncRNA NR_003508, lncRNA uc008scb.1, and lncRNA ENSMUST00000159072) were selected for validation, and their CNC network indicated their involvement in regulating several inflammation and immune-associated pathways in lung damage caused by PM exposure.

m^6^A methylation is the most prevalent RNA modification and plays a crucial role in a variety of biological processes. Accumulating data suggest that m^6^A methylation participates in various lung diseases, such as lung cancer, pulmonary fibrosis, asthma, and acute lung damage [20,21,22]. Teng et al. found that m6A modification promotes immune infiltration and airway Th2 immune response in asthma [20]. Hu et al. showed that m6A modification plays an important role in NSCLC cell proliferation, invasion, and migration [23]. The core mechanism of lung injury caused by PM is inflammation, oxidative stress, and immune response changes [24,25]. Our previous study comprehensively characterized the m^6^A RNA methylome in lung tissues of mice exposed to PM and demonstrated that m^6^A-modified IL-1a plays a proinflammatory role in PM-induced airway inflammation in vitro [11]. Meanwhile, Guo et al. showed that METTL16 regulates the m^6^A methylation modification of sulfatase 2 to promote PM2.5-induced pulmonary microvascular injury in COPD [26]. However, the expression and function of m^6^A-modified lncRNAs in lung damage caused by PM remain unknown. Here, we revealed the comprehensive features of m^6^A-modified lncRNAs in lung tissues of mice exposed to PM.

Several lncRNAs have been identified as vital modifiers of PM-induced lung damage. He et al. discovered that lncRNA MHC-R could regulate dendritic cells immune activity to promote the development of PM2.5-induced COPD [15]. Lee et al. found that another new lncRNA, lnc-IL7R, could combine with enhancer of zeste homolog 2 (EZH2) to inhibit cell apoptosis and senescence and further alleviate the progression of PM2.5-induced COPD [27]. Furthermore, lncRNA TUG1 acts as a competing endogenous RNA for miR-222-3p and enhances airway hyperreactivity (AHR) induced by PM in mice [28]. However, the impact of m^6^A modification on lncRNAs in PM-induced lung damage is not yet understood. Here, we identified three key m^6^A-modified lncRNAs that participated in regulating several inflammation- and immune-associated pathways in PM-induced lung damage using co-expression analysis of lncRNA-seq and RNA-seq data. The upregulated lncRNA NR_003508 in our study was also increased in the lung tissues of mice exposed to LPS and promoted LPS-induced necroptosis in macrophages [29]. These findings suggest that m6A methylation serves as a predominant epigenetic modification of lncRNAs in PM-induced lung damage.

In this study, we show that m^6^A-modified lncRNAs participated in several inflammation- and immune-associated pathways via bioinformatics analysis. Conjoint and co-expression analysis also showed that m^6^A-modified lncRNAs could regulate several key pathways, such as Th17 cell differentiation, ferroptosis, and the MAPK signaling pathway. Sun et al. showed that PM2.5 could increase Th17 cell differentiation to aggravate asthma in an AhR-dependent manner [30]. Xia et al. also found that ultrafine particles could promote Th17 cell differentiation in a Jag1- and Notch 4-dependent manner and exacerbate allergic airway inflammation [31]. Fan et al. demonstrated that PM could induce ferroptosis and activate oxidative stress in lung epithelial cells by activating the Nrf2 signaling pathway [32]. In addition to the studies above, clinical research also demonstrated that PM exposure induces pulmonary injury through activation of inflammatory signaling pathways, including the NF-κB signaling pathway. Fawzy et al. found that exposure to indoor PM in COPD patients could significantly increase the expression of inflammatory factors and oxidative stress [33]. A randomized clinical study with a crossover design by Li et al. found that PM exposure could significantly increase the enrichment of the NF-κB pathway and chemokines, thereby further promoting inflammatory responses [34]. Our previous studies demonstrated that PM could increase the activation of the MAPK pathways to induce airway inflammation. However, no study has revealed the regulatory effects of m^6^A-modified lncRNAs on these PM-related pathways. Here, we demonstrate that m^6^A methylation plays a pivotal role in PM-induced lung damage and that targeting these m^6^A-modified lncRNAs could be a potential therapeutic strategy for PM-induced lung damage.

There are limitations in our study. The mouse model of acute airway inflammation was induced by intratracheal instillation of PM. Compared to the method of inhalation exposure, tracheal instillation does not accurately simulate human exposure processes. Nevertheless, the exposure duration and PM dosage in this study were calculated based on human exposure levels, ensuring that the study results remain valid despite the differences in exposure methods.

## 5. Conclusions

In summary, we revealed the global features of m^6^A-modified lncRNAs in lung tissues of mice exposed to PM using MeRIP-seq. Through bioinformatic analysis, m^6^A-modified lncRNAs were shown to be enriched in several inflammatory, immune, and metabolism-related pathways. Furthermore, three m^6^A-modified lncRNAs (lncRNA NR_003508, lncRNA uc008scb.1, and lncRNA ENSMUST00000159072) were validated using MeRIP-PCR and qRT-PCR, and the CNC network showed that they regulated several inflammation and immune-associated pathways in lung damage caused by PM. The findings of our study have unraveled the functional role of m^6^A-modified lncRNAs in lung damage caused by PM exposure, shedding new light on the regulatory mechanisms underlying PM-induced lung damage.

## Figures and Tables

**Table 1:** The top 10 differentially hypermethylated or hypomethylated m^6^A peaks.

lncRNAs	Regulation	Chromosome	txStart	txEnd	Peak Length	Fold Change	*p* Value
ENSMUST00000135583	up	chr15	79427219	79427400	181	170.6	5.93134 × 10^−13^
uc008juf.1	up	chr2	60369992	60370540	548	157.52	9.23891 × 10^−13^
ENSMUST00000154751	up	chr5	30071281	30071580	299	157	1.29438 × 10^−9^
ENSMUST00000172465	up	chr14	25978576	25978680	104	143.5	2.36309 × 10^−9^
ENSMUST00000175738	up	chr7	15265181	15265620	439	143.5	2.98183 × 10^−9^
NR_046191	up	chr5	38345415	38345493	78	140.1	1.8444 × 10^−9^
NR_038088	up	chr10	79697343	79697350	7	136.7	1.81193 × 10^−9^
ENSMUST00000120341	up	chr4	122205841	122205903	62	136.7	4.27563 × 10^−12^
ENSMUST00000144723	up	chr6	83153701	83154300	599	133.3	1.5141 × 10^−9^
NR_029464	up	chr12	111942281	111942700	419	119.7	1.989 × 10^−11^
ENSMUST00000140004	down	chr7	28086221	28086880	659	305.3	6.90569 × 10^−13^
ENSMUST00000144451	down	chr8	93952946	93953024	78	209.3	4.4487 × 10^−10^
NR_015540	down	chr1	89676821	89677080	259	202.3	1.51477 × 10^−13^
NR_045969	down	chr7	98178421	98178533	112	141.7	3.48445 × 10^−10^
ENSMUST00000152848	down	chr12	56696301	56697200	899	137.9	2.57292 × 10^−9^
ENSMUST00000136726	down	chr14	30002362	30002820	458	132.2	5.80821 × 10^−12^
ENSMUST00000148311	down	chr3	93288941	93289320	379	131.9	2.82892 × 10^−10^
NR_045480	down	chr6	126287781	126288340	559	123	1.70799 × 10^−9^
uc012ejf.1	down	chr6	29799438	29799584	146	119.3	3.08992 × 10^−11^
ENSMUST00000159769	down	chr14	101828967	101829058	91	116.2	8.16182 × 10^−9^

## Data Availability

Data are available upon request.

## Supplementary Materials

The following supporting information can be downloaded at: https://www.mdpi.com/article/10.3390/toxics13020098/s1, Figure S1. Go and KEGG analysis of differentially expressed lncRNAs. (A) GO analysis of differentially upregulated lncRNAs in the biological process category. (B) GO analysis of differentially upregulated lncRNAs in the cellular component category. (C) GO analysis of differentially upregulated lncRNAs in the molecular function category. (D) GO analysis of differentially downregulated lncRNAs in the biological process category. (E) GO analysis of differentially downregulated lncRNAs in the cellular component category. (F) GO analysis of differentially downregulated lncRNAs in the molecular function category. (G) KEGG analysis of differentially upregulated lncRNAs. (H) KEGG analysis of differentially downregulated lncRNAs. GO, Gene Ontology.KEGG, Kyoto Encyclopedia of Genes and Genomes. Figure S2. The GO and KEGG analysis of lncRNA NR_003508, lncRNA uc008scb.1 and lncRNA ENSMUST00000159072-coexpressed mRNAs. (A) GO analysis of lncRNA NR_003508-coexpressed mRNAs. (B) KEGG analysis of lncRNA NR_003508-coexpressed mRNAs. (C) GO analysis of lncRNA uc008scb.1-coexpressed mRNAs. (D) KEGG analysis of lncRNA uc008scb.1-coexpressed mRNAs. (E) GO analysis of lncRNA ENSMUST00000159072-coexpressed mRNAs. (F) KEGG analysis of lncRNA ENSMUST00000159072-coexpressed mRNAs.
