# Supplementary material for: Transcriptome-Wide Analysis of N6-Methyladenosine-Modified Long Noncoding RNAs in Particulate Matter-Induced Lung Injury
Source: Toxics. 2025 Jan 27;13(2):98. doi: 10.3390/toxics13020098 (PMC11860755; doi:10.3390/toxics13020098)
Supplement: Supplementary file 1 [file toxics-13-00098-s001.zip › toxics-3414784-supplementary.pdf]

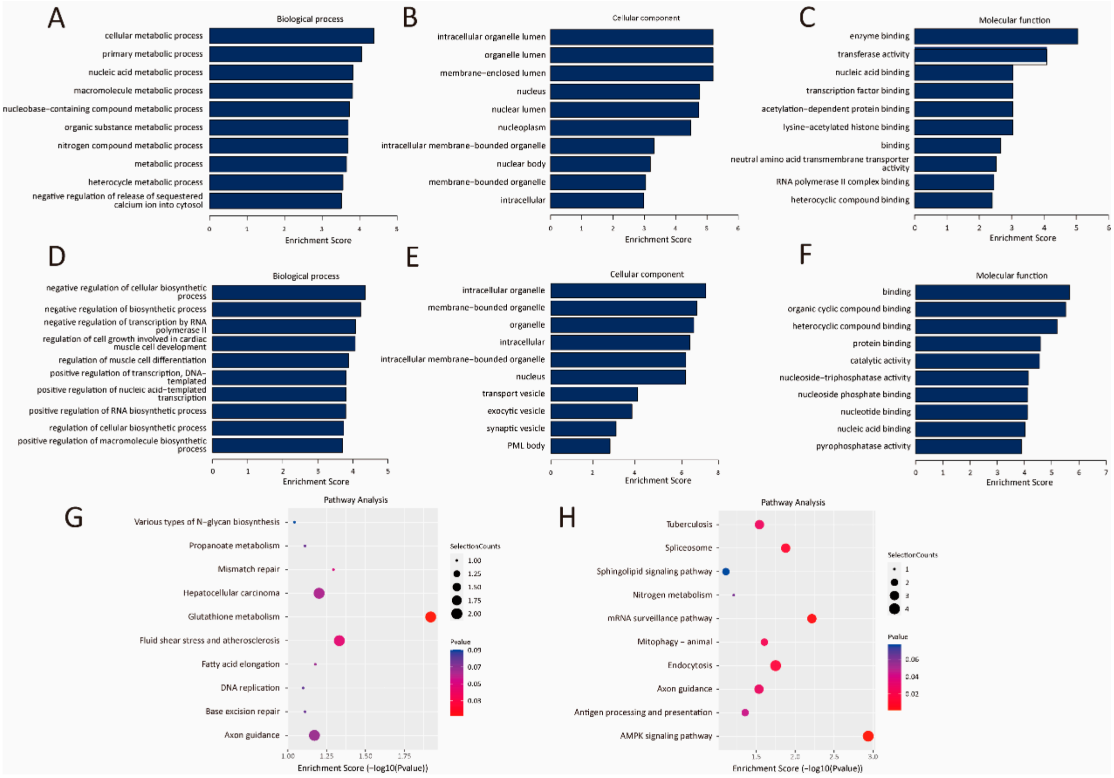

Figure S1. Go and KEGG analysis of differentially expressed lncRNAs. (A) GO analysis of differentially upregulated lncRNAs in the biological process category. (B) GO analysis of differentially upregulated lncRNAs in the cellular component category. (C) GO analysis of differentially upregulated lncRNAs in the molecular function category. (D) GO analysis of differentially downregulated lncRNAs in the biological process category. (E) GO analysis of differentially downregulated lncRNAs in the cellular component category. (F) GO analysis of differentially downregulated lncRNAs in the molecular function category. (G) KEGG analysis of differentially upregulated lncRNAs. (H) KEGG analysis of differentially downregulated lncRNAs. GO, Gene Ontology.KEGG, Kyoto Encyclopedia of Genes and Genomes.

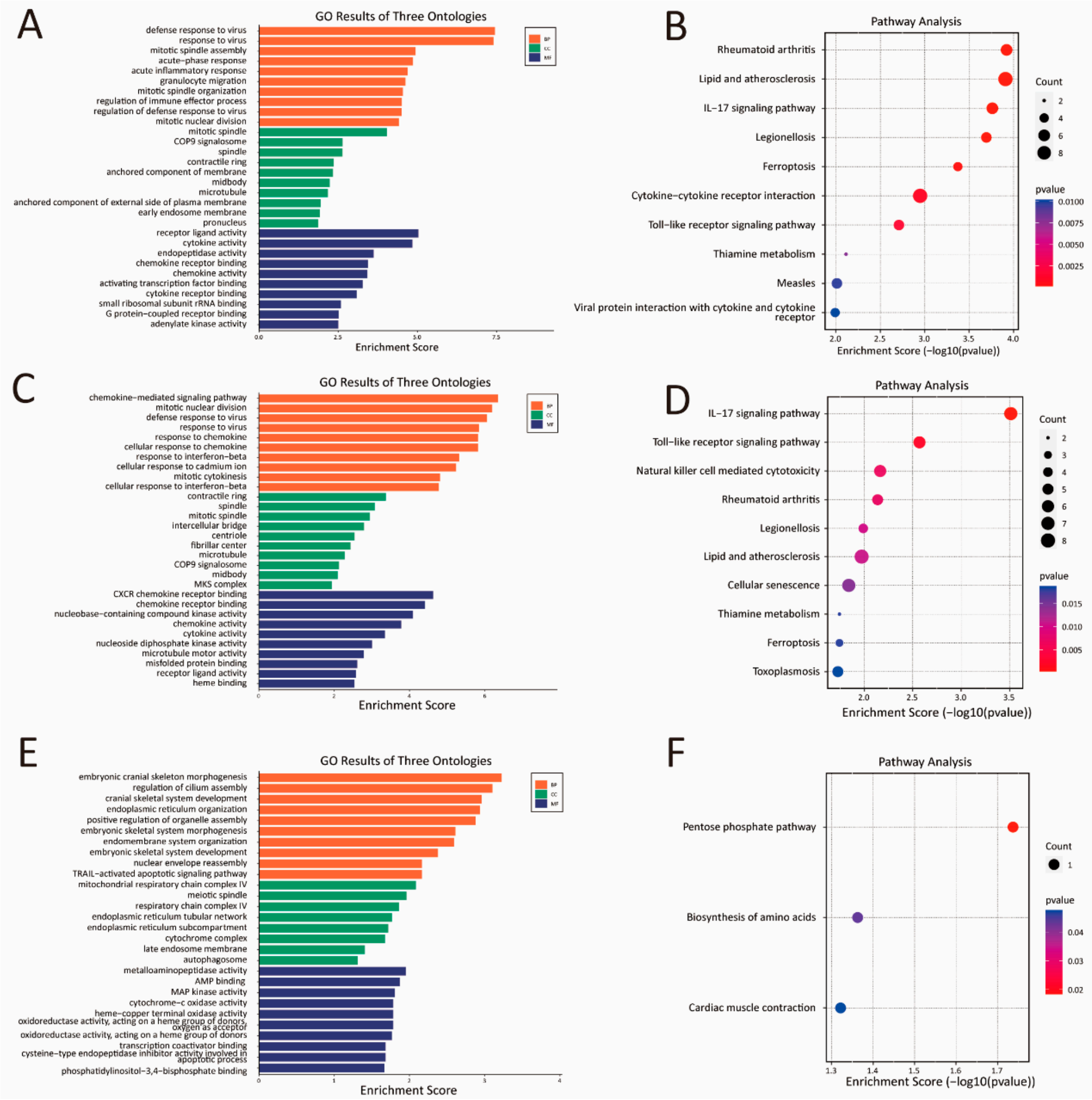

Figure S2. The GO and KEGG analysis of lncRNA NR_003508, lncRNA uc008scb.1 and lncRNA ENSMUST00000159072-coexpressed mRNAs. (A) GO analysis of lncRNA NR_003508-coexpressed mRNAs. (B) KEGG analysis of lncRNA NR_003508-coexpressed mRNAs. (C) GO analysis of lncRNA uc008scb.1-coexpressed mRNAs. (D) KEGG analysis of lncRNA uc008scb.1-coexpressed mRNAs. (E) GO analysis of lncRNA ENSMUST00000159072-coexpressed mRNAs. (F) KEGG analysis of lncRNA ENSMUST00000159072-coexpressed mRNAs.
